# Prediction of 1-Year Activity in Systemic Lupus Erythematosus: Hierarchical Machine Learning Approach

**DOI:** 10.2196/70200

**Published:** 2025-08-22

**Authors:** Livia Lilli, Laura Antenucci, Augusta Ortolan, Silvia Laura Bosello, Stefano Patarnello, Carlotta Masciocchi, Marco Gorini, Gabriella Castellino, Alfredo Cesario, Maria Antonietta D'Agostino, Jacopo Lenkowicz

**Affiliations:** 1 Fondazione Policlinico Universitario Agostino Gemelli IRCCS, Largo Agostino Gemelli, 8, Rome, 00168, Italy; 2 Catholic University of the Sacred Heart, Rome, Italy; 3 Department of Rheumatology, Fondazione Policlinico Universitario Agostino Gemelli IRCCS, Rome, Italy; 4 AstraZeneca, Milano Innovation District (MIND), Milan, Italy; 5 Gemelli Digital Medicine & Health Srl, Rome, Italy

**Keywords:** systemic lupus erythematosus, machine learning, hierarchical model, ensemble approach, explainable artificial intelligence

## Abstract

**Background:**

Systemic lupus erythematosus (SLE) is a chronic disease characterized by a broad spectrum of involved organs, including neurological, renal, and vascular domains, with disease activity manifesting through unpredictable patterns that vary across individuals and over time, making the prediction of activity events particularly challenging.

**Objective:**

This paper proposes a hierarchical machine learning model to predict a 12-month SLE activity, defined as the occurrence of at least one event among SLE hospitalization, new organ-involved domain, and neurological, renal, or vascular manifestation within the following year. At each patient’s visit, the model considers all the features at the current time point, the information about the patient’s clinical history, and about its last 12 months, to predict the outcome for the next 12 months.

**Methods:**

The study cohort consists of 262 patients with at least an outpatient visit and an SLE admission from 2012 to 2020, at the Italian Gemelli Hospital, comprising a retrospective longitudinal dataset of 5962 contacts. The data include demographics, laboratory, clinical features (eg, domain involvements and manifestations), treatments, and pathways (eg, contact types as outpatients, hospitalizations, day hospitals, and visit frequency). The variables consider 3 time ranges: features about the current contact and the last 12 months, and the previous patient’s clinical history. The main model was developed by testing different machine learning approaches within a cross-validation setup. The predicted probability outputs were used in a risk stratification analysis, identifying 3 groups of predictions: strong, moderate, and mild. Mild samples were then passed through a second cascade model. The integration of the main model (applied to strong and moderate samples) with the cascade model (applied to mild contacts) forms our final hierarchical model.

**Results:**

The hierarchical model, resulting from the ensemble of the main random forest and cascade decision tree, demonstrated enhanced performance, increasing the area under the receiver operating characteristic curve from 0.696 (95% CI 0.672‐0.719) in the original main model to 0.743 (95% CI 0.717-0.769), particularly for specific patient characteristics. Through the application of explainable artificial intelligence methods, we also identified the key features that significantly influence the model’s predictions. Among the 185 collected features, 15 emerged as the most impactful, including age at contact, response to therapy modifications, abnormal laboratory tests, and clinical manifestations. This analysis plays a crucial role in enhancing model transparency, which is essential for fostering the adoption of artificial intelligence in health care settings.

**Conclusions:**

Our study introduces an explainable and reliable tool for predicting 1-year SLE activity, supporting physicians with an advanced decision-support system to improve patient management. The model identifies key features that may help characterize patient phenotypes, enabling personalized treatment plans and better outcomes. In addition, the methodology can be generalized for predictive analytics in other chronic autoimmune diseases.

## Introduction

### Background

Systemic lupus erythematosus (SLE) is a complex and heterogeneous autoimmune disease characterized by a high heterogeneity of clinical phenotypes with a wide range of involved organ domains and clinical manifestations. Its nature presents significant challenges in patient management, as activity events can occur unpredictably and may simultaneously impact multiple organ systems [1]. Accurate prediction of SLE activity is crucial for optimizing treatment strategies and improving patient outcomes.

Conventional methods for predicting disease activity often rely on basic statistical and machine learning models [2], considering summary indices such as Systemic Lupus Erythematosus Disease Activity Index (SLEDAI), the principal indicators for the activation of a patient with SLE [3,4].

Furthermore, recent advancements in machine learning offer new opportunities to enhance predictive capabilities by leveraging complex patterns within clinical data. In particular, hierarchical machine learning models have been implemented in various medical applications by combining the strengths of multiple algorithms to improve prediction accuracy [5,6]. In addition, explainable artificial intelligence (XAI) techniques are widely used to analyze the most predictive features [7-9].

**Figure 1.**
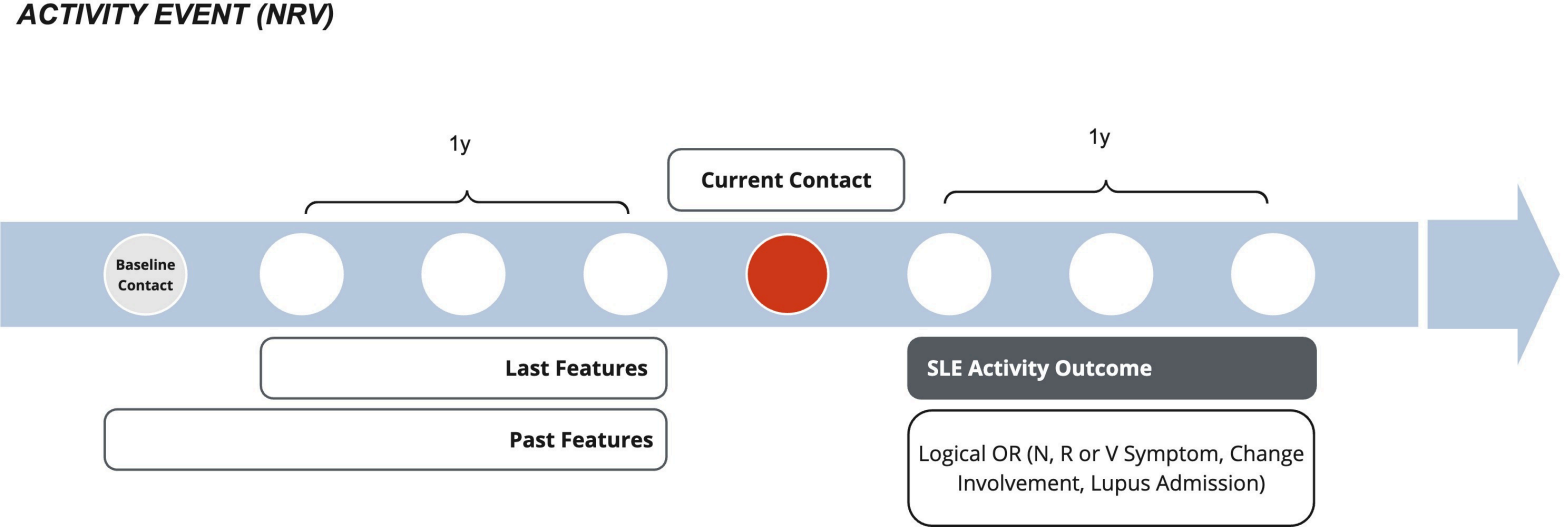
The model predicts the occurrence of a systemic lupus erythematosus activity event for a patient at a certain contact in the next 12 months. For the prediction, it takes as inputs the patient’s features about the current contact, and the information about its clinical history (past features) and the last 12 months (last features). N/R/V: neurologic or renal or vascular.

In the context of SLE, there is growing interest in predictive tools that combine accurate use of longitudinal patient data with clinically interpretable outputs to better support routine decision-making; however, current approaches do not always integrate temporal information effectively or provide transparent reasoning behind predictions. In response to these challenges, this paper introduces a novel hierarchical machine learning model specifically designed to predict the occurrence of SLE activity within a 12-month time frame. The model is based on comprehensive longitudinal data of patients with SLE collected inside the SLE Data Mart of the Italian Gemelli Hospital in Rome, which consists of a comprehensive collection of structured data of patients with SLE, including demographic, laboratory results, treatments, involved domains, and clinical manifestations. At each patient’s contact, the model considers all the features at the current time point, and the information about the clinical history of the patient and about its last 12 months, to predict the outcome for the next 12 months (Figure 1).

The work consists first of developing a main random forest model to categorize patients’ contacts into strong, moderate, and mild risk groups based on prediction confidence. To improve the accuracy of mild predictions, a cascade decision tree model is integrated, whose ensemble generates the final hierarchical model. The model’s performance is evaluated against a testing set, and its predictivity is assessed through metrics, such as the area under the receiver operating characteristic curve (AUC). In addition, we aim to provide physicians with an explainable decision-support tool by identifying key predictive features through XAI techniques, including age at contact, therapy changes, abnormal laboratory values, involved domains, and specific manifestations. The checklist for the guidelines “Machine Learning Predictive Model for Biomedical Data,” which were used to structure and report this study, is provided in Checklist 1.

### Related Works

Several studies explored the implementation of machine learning models in the SLE domain [2]. Among the most inherent to our purpose, there is the approach previously proposed by Ceccarelli et al [10], where SLE-related clinical and laboratory manifestations are used to predict the comprehensive disease control, an outcome based on the achievement of remission and the absence of damage progression in the next time contact. In this work, patients are analyzed along their history, and data are collected starting from the baseline contact, and then every 12 months. In the work of Alves et al [3], the concept of SLE activity (expressed in terms of SLEDAI) is presented. In particular, they proposed a multivariable ordinal regression model for the prediction of disease activity in terms of SLEDAI ranges, for a specific encounter. Also, in this paper, patients have a longitudinal history of contacts. In addition, a real-world dataset of clinical reports was used, where the explanatory features were extracted and used as inputs to the model. Furthermore, Hoi et al [4] developed a ranking model for the prediction of the high disease activity outcome, based on the SLEDAI ranges. In this case, demographic and laboratory variables were used as inputs.

The study by Reddy and Delen [11] proposed a model for predicting the hospital readmission of patients with SLE in 30 days, by using deep-learning models for extracting longitudinal relationships inside electronic health records (EHRs). Furthermore, the study of Ceccarelli et al [12] worked on the prediction of the chronic damage defined in terms of the SLICC/ACR Damage Index, through the usage of Recurrent Neural Networks. The paper by Chen et al [13] is instead about the development of a machine learning model for the prediction of renal flare 5 years after remission, with the integration of a risk stratification model. Finally, a more recent study by Touma et al [14] shows the implementation of historical models for long-term outcomes to identify predictors of disease activity, flare rate, glucocorticoid use, organ damage, and mortality beyond the first year of treatment.

There are a few other studies that implemented machine learning and deep learning approaches for prediction modeling in the rheumatologic domain [15-18]. While for the implementation of XAI in the analysis of features’ impact, there are several recent papers [19,20], where Shapley Additive Explanations (SHAP) [21] module is integrated in the predictive flow for the prediction of clinical outcomes.

## Methods

### Data Collection

The SLE data mart of the Italian Gemelli Hospital was used as an input dataset for this study. It consists of an extensive collection of structured information about patients with SLE observed along their longitudinal history at the Gemelli Hospital. The data mart considers a cohort of 262 patients with at least an outpatient visit and a hospitalization filtered by the *ICD-9* (*International Classification of Diseases, Ninth Revision*) codes 710.0 and 695.4, selected based on having had at least 1 such admission between 2012 and 2020, and with a minimum of 1 year of follow-up. Although the selection window is 2012‐2020, the complete dataset spans a broader range from 2005 to 2022, to capture the full longitudinal clinical history available for each patient. The baseline is defined as the first recorded contact at the hospital, within this extended time frame.

In this cohort, 88% (230/262) of patients are female, with an age at baseline of 43 years, a follow-up of 6 years, and 16 contacts, in terms of median (Table 1). Patient observations occur during outpatient visits, hospital admissions, and day hospitals, for a total of 5962 contacts. Data are about demographics, treatments, clinical manifestations, involved domains, and laboratory and come from both structured and unstructured data sources. Data mining and natural language processing techniques were used for extracting and managing all the information coming from structured and unstructured sources, formatting them into the final SLE data mart (details about the NLP methodology can be found in our previous work [22]).

As an example of how variables were extracted, there are the organ domain involvements, the key features that primarily characterize patients with SLE. They are extracted through NLP from the textual EHRs, where the physician in charge usually reports the SLE diagnosis, specifying the organs affected. Once an organ involvement is recorded, it is considered persistent over time, as it reflects the chronic nature of organ impact from lupus for that specific patient. In our retrospective dataset, we distinguish among 8 different organ domains: articular, cutaneous, hematological, neurological, renal, vascular, serosal, and systemic. We define a “new involvement” as the first occurrence of an organ being involved that was not previously documented in the patient’s medical history. This allows us to accurately capture the progression or change in disease manifestation over time.

**Table 1.** Characteristics of the study cohort. For categorical variables, occurrences in absolute and percentage format are reported, while for the numerical variables, median is shown with first and third quartiles.

Characteristic	All patients (N=262)
Female, n (%)	230 (88)
Age at Baseline, median (IQR)	43 (33-54)
Follow-up (y), median (IQR)	6 (3-8)
Contacts (all types), median (IQR)	16 (10-24)
Admission contacts, median (IQR)	1 (0-2)
Outpatient contacts, median (IQR)	12 (7-19)
Day hospital contacts, median (IQR)	0 (0-2)

### Missing Data

During data processing, the issue of missing values affected 2 types of variables: treatments and laboratory data. To address these gaps, we adopted different approaches for each variable type to ensure data consistency and completeness. For laboratory data, missing values are not directly reported as numerical values in the input dataset. Instead, 2 binary variables are used to represent each laboratory measurement: “normal range” and “out of range.” When values are missing, both variables are automatically set to “0,” indicating the absence of recorded information. In such cases, there is no recorded evidence to determine whether the laboratory value was normal or out of range. For treatment data, missing therapies are imputed with the therapy information from the previous visit. This approach reflects the clinical practice of assuming continuity of therapy unless explicitly stated otherwise.

### Outcome Definition

The hierarchical model aims to predict the occurrence of an activity event in the following 12 months of a patient with SLE. The activity event is a binary variable, and it was defined through a logical OR combination of the 3 main events that represent a progression or a clinically relevant modification in SLE: (1) the manifestation of a symptom in the neurologic, renal, or vascular domains [23]; (2) the addition of a new involved domain; and (3) a hospitalization filtered by *ICD-9* codes 710.0 and 695.4. To enhance the relevance of the outcome, hospitalizations were further restricted to those occurring in departments more closely associated with SLE disease activity (ie, hematology, nephrology, dermatology, neurology, and angiology), excluding less relevant units such as infectious diseases and orthopedics. In addition, hospitalizations for continuation of cyclophosphamide or rituximab treatment were excluded, while only the first infusion was considered an activity event due to its typical association with high disease activity.

Then, if a patient presents at least 1 of the above events in the next 12 months with respect to the current observational contact, then the patient is labeled as positive to the outcome for that current time point. Of the overall dataset, 48% (2828/5962) of samples show a positive activity outcome. The prediction is performed independently at each contact, treating each visit separately while simultaneously building the longitudinal trajectory of the patient. This approach allows the model to generate multiple predictions across different time points for the same patient, effectively capturing the entire longitudinal history and enabling continuous monitoring of disease activity.

### Input Features

The implementation of the hierarchical model considers an initial set of 181 SLE features available from the SLE data mart. The scope was to consider all the variables that clinically describe the patient, by providing SLE information about the current contact (current features), the clinical history (past features), and the last 12 months (last features). A feature engineering process was implemented in order to make all the variables processable by the model. Then all the features are binary or integers, depending on the type of information. A detailed guide on features’ descriptions and types is provided in Multimedia Appendix 1, while preliminary statistics for all the variables can be found in Multimedia Appendix 2.

A univariate feature selection was performed in order to keep just the most significant features compared to the outcome for the model training. We leveraged the chi-square and the Mann-Whitney *U* tests for categorical and numerical variables, respectively. At the end of the statistical analysis, we kept all the features with a significant *P* value under .05, using them as inputs to the model for training and inference.

### Main Model

The predictive model consists of the implementation of a Grid Search algorithm over multiple machine learning models, that are, logistic regression, decision tree, random forest, and XGBoost. At each iteration for a specific model, the algorithm tests several combinations of hyperparameters, performing a k-fold cross-validation, with 5 folds. At the end of the process, the model with the set of hyperparameters with the highest AUC performance was selected as the main model for this study. This method ensures that the chosen model is not only optimal but also robust and generalizable to new, unseen data.

Only 30% of patients were used as the testing set for the inference phase, while the remaining 70% represents our training set. Then contacts of a patient all belong to the same split set. The splitting was implemented randomly, by verifying two conditions: (1) that the outcome was balanced over both train and test sets, (2) and that patients were represented in a balanced way across the sets (ie, we ensure that 70% of training patients corresponds to 70% of contacts approximately). The random split was repeated until both the above conditions were met. We then got a training set of 183 patients and 4117 contacts, and a testing set of 79 patients and 1845 contacts. Furthermore, for construction, the last and past features are null at the patient’s baseline. We then removed the first contact of each patient from the training set to prevent the model from learning biases caused by these zero values, obtaining a training set of 3934 samples. This ensures the model focuses on meaningful patterns rather than the artificial absence of data in early contacts.

For the implementation, we used the Scikit-Learn Python library [24]. The results of the main model were analyzed in terms of features’ explainability through XAI techniques, for debugging purposes. To this scope, the SHAP module was used [7].

### Risk Stratification

The output probabilities of the main model were used to define the risk of a patient at a specific contact. Based on the positive or negative outcome, we defined 2 risk categories, high and low. Each category is then distinguished as strong, moderate, or mild, based on the predicted probability (PP) value: when it is very close to the prediction cutoff, the risk category is mild, and it tends to become moderate and then strong, moving away from the threshold.

### Cascade Model

The previously described risk stratification was essential to identify patient subgroups at a given contact, categorizing them into high- and low-risk levels with varying degrees of predictability, ranging from mild to moderate and strong, based on prediction confidence. Mild contacts represent cases where the main model predicts the outcome with greater uncertainty due to a lack of sufficient discriminant information to make a confident decision. To enhance the overall performance, a cascade model was then ensembled, specifically trained on mild-risk samples. In agreement with physicians, a smaller set of features was used as inputs, such as demographic information about gender and age at contact and current treatments not used in the main model. We then trained a decision tree machine learning algorithm to get a tree of rules to be applied on the uncertain samples. From the resulting tree, we found the branches with the highest accuracy rate, and we used them to build up a rule-based algorithm to be applied after the main model, just on the mild-risk contacts. The integration of both models, the main model applied to strong and moderate samples, and the cascade model applied to the mild samples, results in our final hierarchical model.

### Ethical Considerations

The study conforms with European General Data Protection Regulation directives and consequent Italian legislation references (European Union Directive 2016/679 and under Italian Laws: Decreto Legislativo 196/2003, Decreto Legislativo 101/2018, and Autorizzazione Generale Garante 9/2016). In line with the General Data Protection Regulation directives and the designation of Policlinico Gemelli Hospital as National Center for Health Research and Care from the Italian Ministry of Health, observational studies do not require a specific request for patient consent. All technical procedures and security measures have been reviewed and approved in the context of Data Protection Impact Assessment from the hospital Data Privacy Unit and Data Protection Officer. The study protocol conforms to the ethical guidelines of the 1975 Declaration of Helsinki and has received approval from the Policlinico Gemelli Ethics Committee (Comitato Etico Territoriale Lazio Area 3, protocol 0034012/22).

## Results

### Feature-Based Explainability Analysis

For each contact, we considered a set of features regarding the current status of the patient (current features), the events of the last 12 months (last features), and its clinical history (past features), for a total of 181 variables. A selection algorithm was applied to the training set, and 125 variables resulted significantly associated with the activity outcome (*P*<.05). Given the large number of variables, only the most representative ones are highlighted for a preliminary overview (Table 2). Among the significant features, younger age at contact is strongly linked to a positive activity outcome in the next 12 months. Furthermore, the addition of a new organ involvement emerged as a critical indicator of future disease activity. Also, the temporal delta between contacts, which calculates the number of days since the previous visit, is significantly different; it indicates more frequent visits for patients with an activity event, making it a useful variable to account for irregular follow-up patterns. Finally, the treatment pathways, expressed in terms of therapy changes, show a strong correlation with the outcome; both the presence of a step-down therapy change in the patient’s history and the frequency of step-down therapy changes are significantly associated with disease activity. These findings underline the importance of identifying key clinical predictors to improve outcome monitoring.

The selected features were then given as input to the model, for the training and inference phases. During the model development, we applied XAI techniques to analyze features’ importance in our predictive main model. To this scope, we leveraged the SHAP Python module, which quantifies the impact of each feature on the model’s predictions, providing insights into the contribution and interaction of features with respect to the outcome.

**Figure 2.**
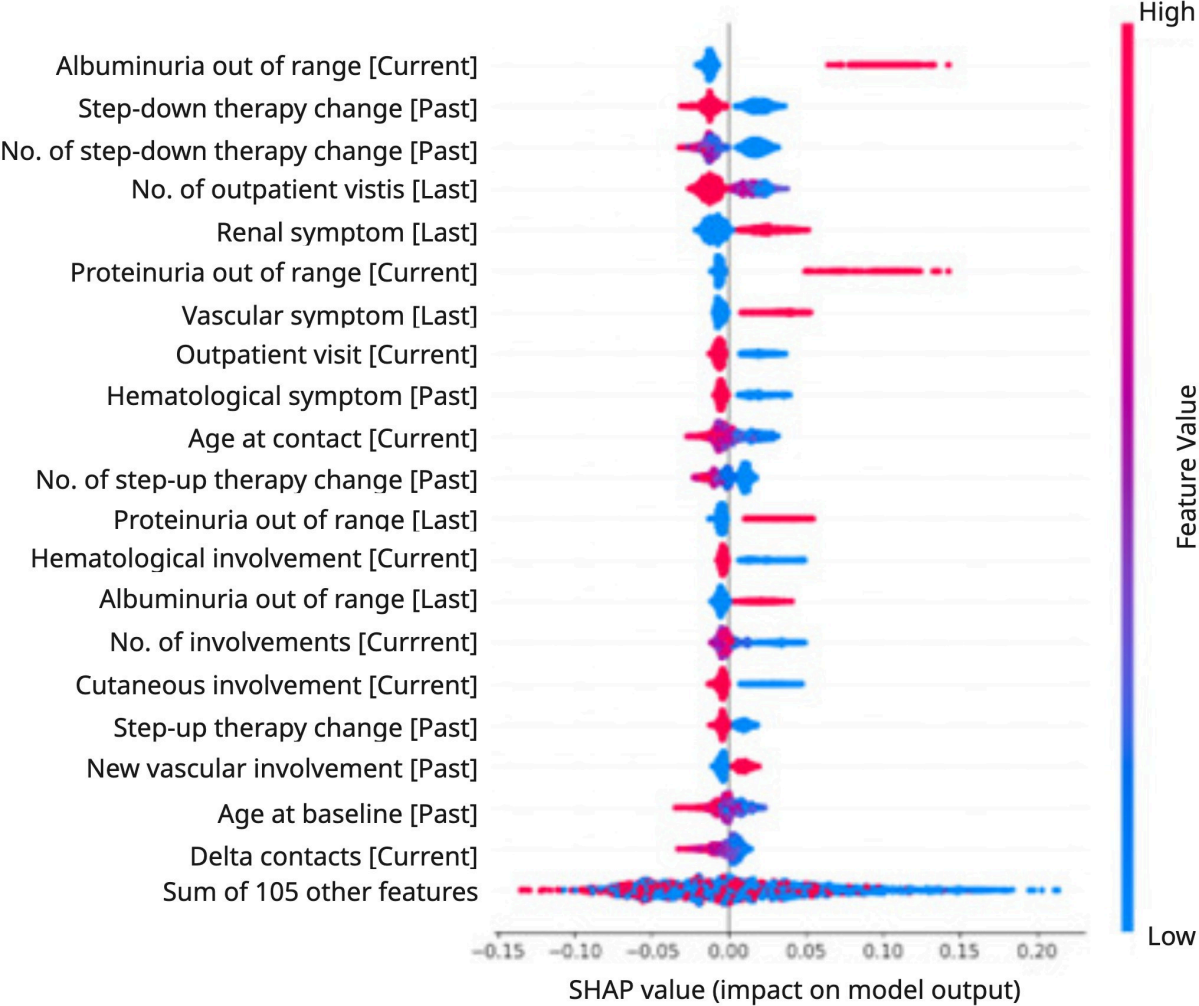
SHAP Beeswarm plot describing impact and direction of each feature on the outcome prediction, ordered by importance. SHAP: Shapley Additive Explanations.

The Beeswarm plot was generated by the model during the outcome prediction over the testing contacts (Figure 2). It represents both the distribution and magnitude of feature effects, highlighting the features with the most significant impact and their direction (positive or negative) on the model’s output. As an example, for young patients, the model prediction tends to be positive to an activity event. The blue data points correspond to low values of the age at contact variable, and they are all distributed on the right side of the y-axis, with positive SHAP values.

**Figure 3.**
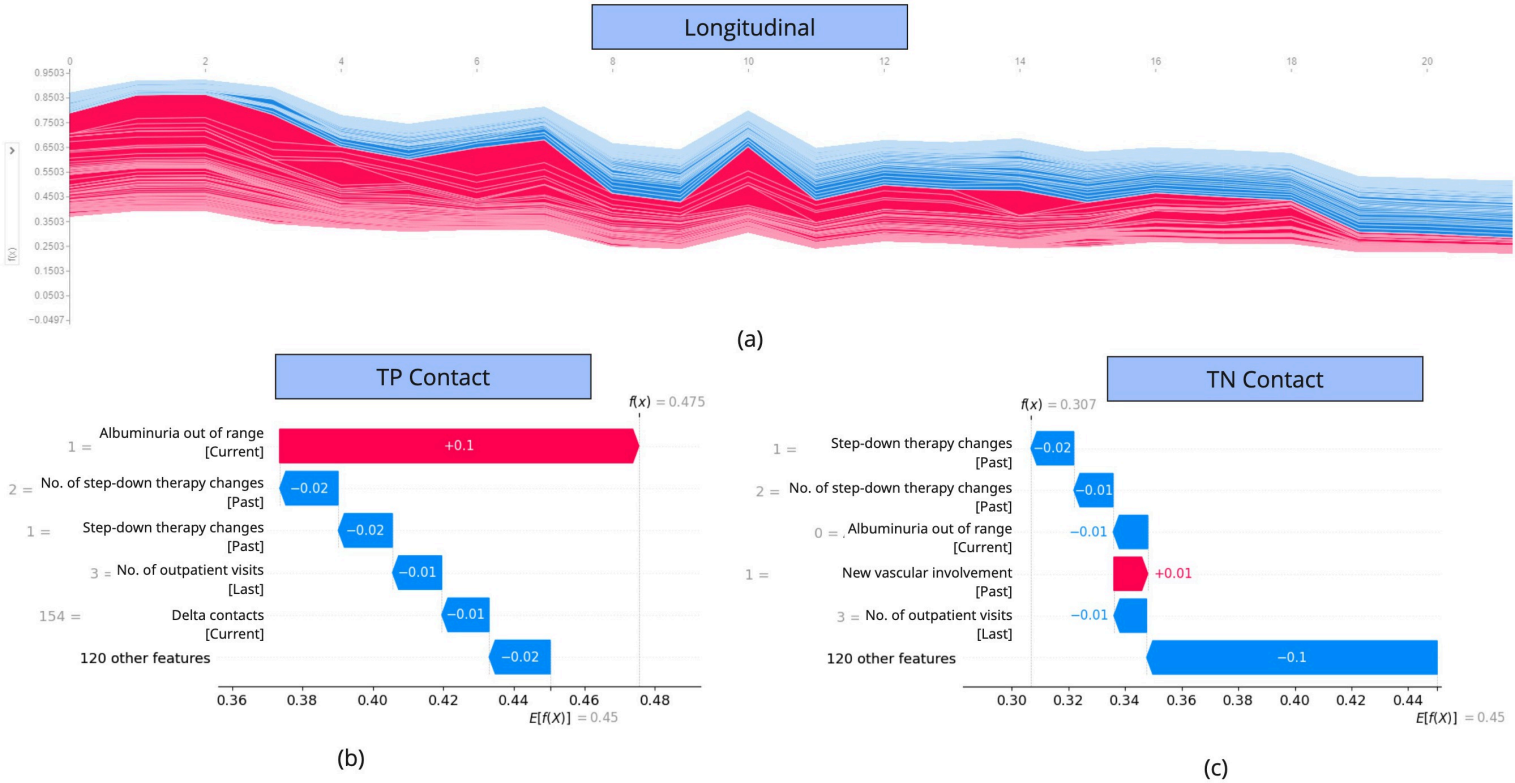
Longitudinal SHAP analysis for a specific patient. The top force plot shows the aggregated impact of the input features on the patient’s prediction over time. At the bottom, the most impactful features for 2 contact visits are shown. In particular, the 2 samples present a true positive prediction (bottom-left) and a true negative prediction (bottom-right). TP: true positive; TN: true negative.

We also conducted a SHAP longitudinal analysis on the individual patients, through the Force plot, where the aggregated predictive impact of the features over the patient’s longitudinal history is displayed (Figure 3). Given the longitudinal representation of a patient, we then conducted an in-depth analysis of each time point, looking at the detailed impact of each feature on the related prediction. In Figure 3, true positive prediction is reported, where the model tends to predict the presence of an activity outcome in the following 12 months, mostly based on the out-of-range albuminuria. Also, a true negative contact is shown, where the step-down therapy change pushes the prediction to be negative, together with normal albuminuria and the high number of outpatient visits in the last year.

**Table 2.** Feature selection: list of the most representative features of the 125 selected by the algorithm. For categorical variables, occurrences in absolute and percentage format are reported, while for the numerical variables, median is shown with first and third quartiles.

Feature	Activity event (n=1772)	No activity event (n=2162)	*P* value
Current contact			
Age at contact, median (IQR)	44.0 (36.0-53.0)	46.0 (37.0-55.0)	<.001
Outpatient visit, n (%)	1217 (68.68)	1779 (82.28)	<.001
Day hospital, n (%)	413 (23.31)	331 (15.31)	<.001
Admission, n (%)	152 (8.58)	57 (2.64)	<.001
Delta contacts, median (IQR)	77.0 (33.0-136.0)	90.0 (32.0-153.0)	.044
New involvement, n (%)	204 (11.51)	113 (5.23)	<.001
Last 12 months			
Total of outpatient visits, median (IQR)	2.0 (1.0-3.0)	3.0 (2.0-3.0)	<.001
Outpatient visit, n (%)	1370 (77.31)	1852 (85.66)	<.001
Day hospital contact, n (%)	509 (28.72)	482 (22.29)	<.001
Admission, n (%)	369 (20.82)	262 (12.12)	<.001
New involvement, n (%)	445 (25.11)	369 (17.07)	<.001
Total of new involvements, median (IQR)	0.0 (0.0-1.0)	0.0 (0.0-0.0)	<.001
History			
Outpatient visit, n (%)	1526 (86.12)	2018 (93.34)	<.001
Step-down therapy change, n (%)	832 (46.95)	1397 (64.62)	<.001
Total of step-down therapy changes, median (IQR)	0.0 (0.0-1.0)	1.0 (0.0-2.0)	<.001

### Risk Stratification

**Figure 4.**
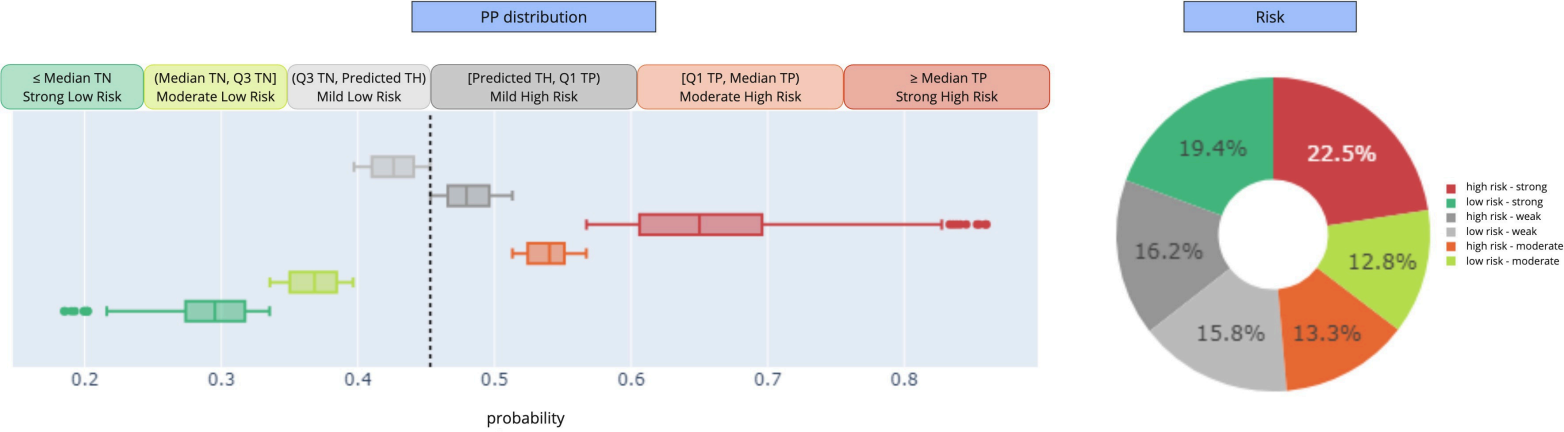
Risk stratification thresholds (left) and risk distribution over the testing contacts (right). PP: predicted probability; TH: threshold; TN: true negative; TP: true positive; Q1: first quartile; Q3: third quartile.

The risk stratification was performed considering the main model’s PP values. In particular, high and low categories are distinguished by the prediction cutoff automatically computed during the training phase, which equals 0.45. For samples predicted with an activity event in the next 12 months (then with a PP>0.45), the risk is high; otherwise, the risk is low. Then, the level of predictability is chosen between strong, moderate, and mild, depending on whether the PP is far or near the prediction cutoff. For this purpose, we used the PP distribution. For high-risk predictions, we used the median and the first quartile of the true positives as thresholds, while for low-risk predictions, we relied on the median and the third quartile of the true negatives (Figure 4). From this, we got 32% of mild contacts (ie, 590 samples), where the model could not predict the outcome with a sufficiently high predictability (Figure 4).

### Hierarchical Model

The main model consisted of the machine learning algorithm, which returned the best AUC performances over the 1845 test samples, where random forest achieves an AUC=0.696, versus logistic regression (AUC=0.64), decision tree (AUC=0.653), and XGBoost (AUC=0.693). The hierarchical model was then built based on the combination of the main model with a cascade model applied over the mild-risk contacts. In particular, we trained a decision tree model on the activity outcome, over a training set of 545 mild contacts (we considered the same risk stratification over the training set), keeping the same split used in the main model. We then obtained the tree of rules applied over the evaluation set of 590 contacts. From this, we then focused on the branches with higher prediction accuracy (over 60%) and clinically approved by the physicians, considering these predictions as more confident (Multimedia Appendix 3). In particular, in these decisional paths, we found that male patients with an age between 31 and 71 years, not treated with glucocorticoids, can be considered as having a negative outcome. Furthermore, female patients in the age range of 31‐64 years, who were treated only with antimalarials, also show a negative activity outcome. Then, for the subset of patients following the above rules, we could associate a low risk with high reliability.

**Figure 5.**
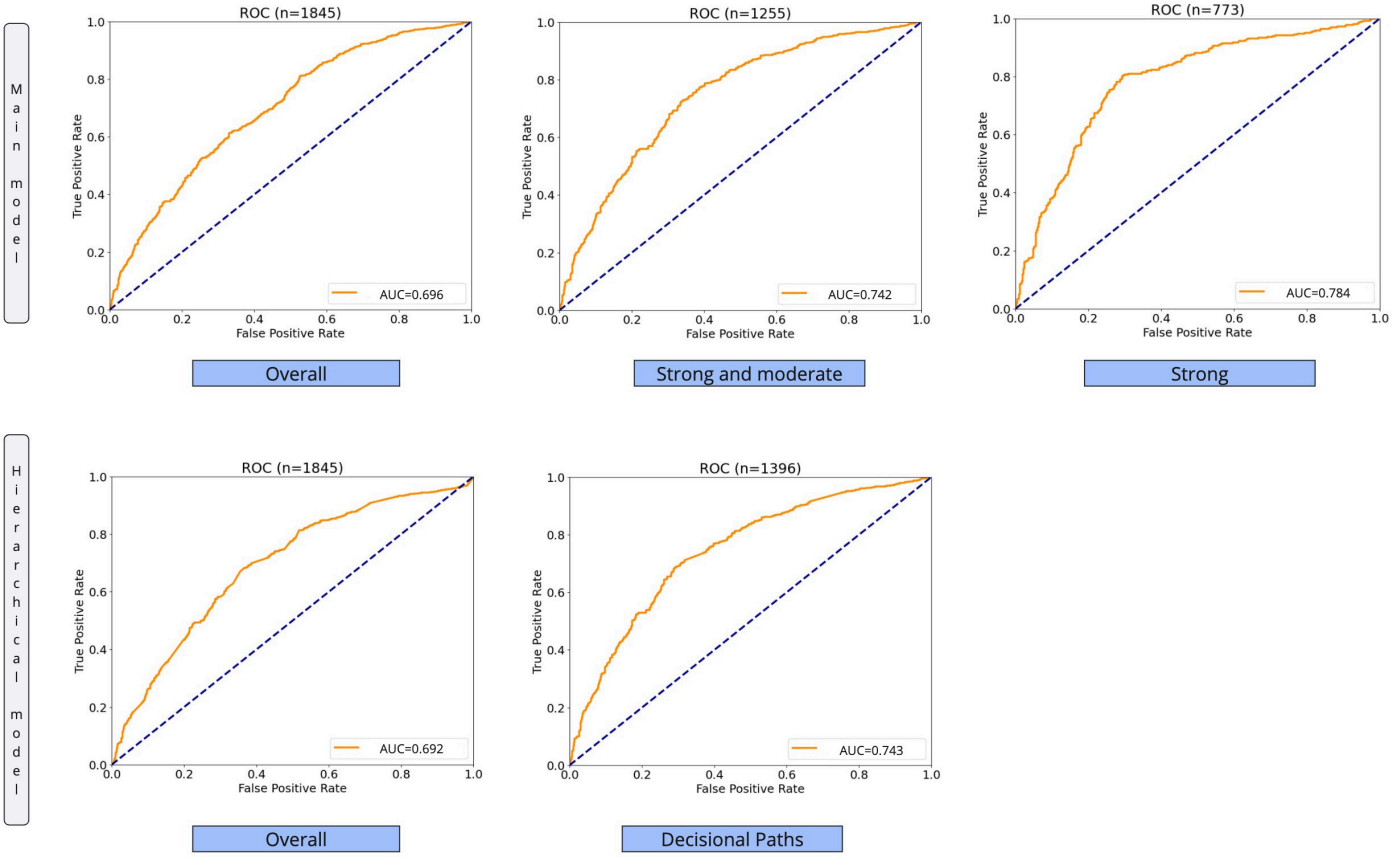
Comparison of results. The first row shows the main model applied overall the dataset, on the strong and moderate contacts, and on the strong contacts. The second row is about the ensemble's results. In the bottom-left, the main model is applied over the strong and moderate contacts, with the cascade model implemented over the mild contacts; in the bottom-right, only the most accurate decisional path of the cascade model is considered. ROC: receiver operating characteristic curve.

The main model’s performance in terms of receiver operating characteristic curve and AUC metric is shown (Figure 5) when applied overall to the 1845 testing samples, with the exclusion of the mild predictions and only over the strong-risk contacts. The model then passes from AUC=0.696 (95% CI 0.672‐0.719; number of contacts=1845) overall the test set, to AUC=0.742 (95% CI 0.715‐0.769; number of contacts=1255), and AUC=0.784 (95% CI 0.752‐0.817; number of contacts=773) when considering both moderate and strong contacts, as well as strong contacts only. Finally, the hierarchical model results are reported for overall the dataset and considering just the most accurate decisional paths, with AUC=0.692 (95% CI 0.667‐0.716; number of contacts=1845) and AUC=0.743 (95% CI 0.717-0.769; number of contacts=1396), respectively.

## Discussion

### Principal Results and Comparison With Previous Work

In this paper, we proposed an ensembled approach where multiple models are combined to predict the activity event of a patient with SLE in the next 12 months.

In literature, several works are related to the development of SLE predictive models [2-5], exploring the problem from multiple perspectives. These studies vary in terms of predicted outcomes, such as remission, chronic damage, or hospital readmission, and leverage different types of input data, including clinical manifestations, laboratory values, and treatments. Some also focus on modeling disease progression over time. Taken together, these studies have contributed valuable evidence to advance personalized diagnosis and treatment in SLE.

Our purpose consists of presenting a novel approach, developing a hierarchical model, that integrated with risk analysis and XAI techniques, returns a 1-year prediction of the SLE activity outcome. The definition of an activity event was established by our team of physicians, drawing on their clinical expertise and existing literature [23]. It consists of a combination of the most severe flare types and other severe events (such as hospitalization and new involved organs), identified from both clinical notes and structured data sources. This choice differs from the outcomes used in past works, such as comprehensive disease control, hospital readmission, and chronic damage [3,4,10-18].

Our main model was trained over a set of 125 features, automatically selected based on their significance to the outcome (Table 2). Features are about demographics, clinical manifestations, involved domains, laboratory, and therapy changes, mostly following the information used in the related previous cited works. However, in our case, the same information is reported for the current contact, the last 12 months, and the clinical history. Thus, we allow traditional machine learning models such as random forest to capture both the present and the past of a patient for each observed time point. This setup allows us to treat each contact independently, then conduct a contact-level prediction. In addition, this feature framework, based on a real-world data collection from standard clinical practice, yields a set of predictors that are operationally accessible, making the model potentially replicable across different health care settings. As a future direction, this approach could be integrated with methodologies based on prospective data collection (eg, studies by Ceccarelli et al [10,12]) or derived from public datasets (eg, study by Reddy and Delen [11]) to further improve generalizability and support broader validation.

In the features’ explainability analysis, we used the Python SHAP module in order to deeply analyze the features’ impact during the prediction of the main model. This approach can be particularly useful in real-world clinical practice, as it provides a transparent understanding of the model’s reasoning, supporting informed decision-making through an explainable and reliable tool. Then, we generated the SHAP Beeswarm plot (Figure 2), where the most predictive features are reported, with their impact on the activity event outcome. Many of the findings are already noted in the SLE literature, or derive from the outcome definition, such as the positive impact of proteinuria, albuminuria, young age, and renal symptoms [25,26]. Indeed, the plot shows that albuminuria and proteinuria out of range, the presence of renal symptoms, and young age push the model to predict an activity event for the patient in the following 12 months. However, we also got additional evidence related to the effect of therapy changes, outpatient contacts, temporal distance among contacts, and hematological and cutaneous involvements. Indeed, the plot shows that a high number of outpatient visits (as opposed to inpatient or day hospital admissions) in the last 12 months is associated with a protective effect on the outcome. This suggests that closer clinical monitoring may contribute to a better outcome, whereas inpatient or day hospital admissions could indicate a disease flare or the need for ongoing inpatient treatment. Furthermore, when no organs are involved in the hematological and cutaneous domains, the patient is more likely to have a positive outcome. Conversely, the longer the interval between contact visits, the higher the probability of remaining stable in the following 12 months. Finally, a step-down change in therapy in the past (such as the discontinuation of a biological immunosuppressant) is protective for a positive outcome. Our results are consistent with previous studies, particularly the studies by Ceccarelli et al [10,12], which also highlight hematological manifestations and renal symptoms as key features associated with increased severity, even though they rely on different sets of variables. In addition, a study by Reddy and Delen [11] identifies the time interval between hospital contacts as a predictive factor, but in this case against the risk of rehospitalization.

The risk stratification was then performed on the main model predictions, returning 32% mild-risk contacts (Figure 4), which we used to develop a cascade model. Then, the integration of the main model implemented over the strong and moderate predictions, with the cascade model applied over the mild samples, generated the final hierarchical model. The hierarchical model achieves an AUC of 0.743 when considering the samples following the most decisional paths. In terms of metric value, this result is lower than the AUC=0.784 of the main model applied over the strong contacts, and not so far from the AUC=0.742 of the main model implemented on the moderate and strong samples (Figure 5). However, the hierarchical model, with an almost high metric value, can cover a larger portion of predictable input data.

Results we achieved in terms of AUC reflect the current state of the art given by the previous works [10-12], where the implementation of predictive modeling on SLE using input features similar to ours generated an AUC between 0.70 and 0.77. In the works of Alves et al [3] and Hoi et al [4], results are higher, with metrics equal to 0.90 and 0.829, respectively. However, our variety of input features is larger; in the study by Alves et al [3], textual EHRs are processed through text mining techniques for extracting SLEDAI information, while in the study by Hoi et al [4], laboratory measures and demographic information are considered. Nevertheless, they could serve as inspiration for future work, following their strategy of using SLEDAI ranges as activity indicators, which we could not implement in this study due to the incomplete availability of SLEDAI information at our hospital. While SLEDAI is a widely recognized marker of disease activity, its predictive value for severity is not always consistent [3]. Therefore, further exploration of its role and potential integration with alternative indicators could be a valuable direction for future research.

### Limitations

Our study was conducted on a filtered SLE cohort of the Gemelli Hospital, selecting only patients who had had at least an outpatient visit and an SLE hospitalization between 2012 and 2020, with a minimum of 1 year of follow-up. This approach inherently introduces a selection bias, as our model is trained on a cohort representing more severe cases. Future work could address this limitation by applying our model to a broader cohort that also includes outpatient-only cases, thereby reducing the current selection bias. In addition, extending our research to a multicentric scenario would be valuable for assessing the model’s generalizability across different patient populations.

### Conclusions

This study aims to provide a hierarchical machine learning approach, where the ensemble of multiple architectures and the usage of XAI tools and risk stratification analysis contribute to the development of an explainable model for the prediction of SLE activity in the next 12 months that could support physicians to modify the therapeutic approach.

Relevant features identified through the learning process are of different kinds, that are (1) demographics (eg, risk of severe events is reduced for older patients), (2) clinical (as an example, occurrence of renal symptoms in the past 12 months increases risks), and (3) treatment related (eg, patients who have shown good response to a therapy change from biological to conventional in their disease history are less exposed to severe event occurrence). From a predictability analysis, we found that our approach can achieve an AUC of 74.3% when applied under specific patients’ characteristics. The results mostly reflect other related works in literature, always in the domain of SLE predictive modeling, but with the integration of an XAI tool, which makes the predictions explainable at feature level.

In conclusion, our hierarchical model offers accurate 12-month activity predictions along with an explainable framework for result interpretation. This contributes meaningfully to predictive analytics in chronic autoimmune diseases, paving the way for future studies to build on our findings and advance patient care.

## Supplementary material

10.2196/70200 Multimedia Appendix 1 Detailed guide on input features, providing an overview of variable macro-categories, a comprehensive list of all features with definitions, types, and formats, and clear explanations of key concepts such as symptoms, flare, and complexity.

10.2196/70200 Multimedia Appendix 2 General statistics of the input features about the current contact, the last 12 months, and the clinical history.

10.2196/70200 Multimedia Appendix 3 Decisional paths of the cascade decision tree model, with the highest accuracy rate.

10.2196/70200 Checklist 1 Checklist for Machine Learning Predictive Model for Biomedical Data guidelines.

## Abbreviations

AUC: area under the receiver operating characteristic curve
EHR: electronic health record
*ICD-9*: *International Classification of Diseases, Ninth Revision*
PP: predicted probability
SHAP: Shapley Additive Explanations
SLE: systemic lupus erythematosus
SLEDAI: Systemic Lupus Erythematosus Disease Activity Index
XAI: explainable artificial intelligence
