# Supplementary material for: Prediction of 1-Year Activity in Systemic Lupus Erythematosus: Hierarchical Machine Learning Approach
Source: JMIR Form Res. 2025 Aug 22;9:e70200. doi: 10.2196/70200 (PMC12373299; doi:10.2196/70200)
Supplement: Multimedia Appendix 1 [file formative-v9-e70200-s001.docx]

# Multimedia Appendix 1

This appendix provides a comprehensive explanation of the input variables used during model development. Specifically, Table 1 offers an overview of the main feature categories extracted from clinical reports. These categories include Involvement, Symptom, Flare, Laboratory, Complexity, Therapy Change, and Treatment, each representing distinct aspects of patient data. The features are derived from both structured and unstructured clinical information, with categorizations established according to clinical practice and expert guidelines. For a detailed breakdown, Table 2 presents the complete list of variables, including a brief definition, the variable format (numerical or categorical), and the range type (indicating whether they pertain to the current contact, the last 12 months, and/or the patient’s history). Furthermore, Tables 3, 4 and 5 outline the criteria used to compute Symptom, Flare and Complexity features. These guidelines have been carefully developed by our clinical team and are further analyzed in terms of patient pathways and clinical implications in a separate study, currently under publication.

Table 1. Overview of the main feature categories used in model development, representing distinct aspects of patient data derived from both structured and unstructured clinical information.

| **Variable Category** | **Description** |
| --- | --- |
| *Involvement* | Extracted through NLP from the anamnestic section of clinical reports, where the specific type of Lupus involvement is explicitly documented.  Once an involvement is identified, it is considered persistent over time, reflecting the chronic nature of the condition. |
| *Symptom* | Extracted through NLP from the objective examination sections of clinical reports and combined with laboratory values, to be categorized into 8 domains as defined by the clinical team. |
| *Flare* | Defined over a set of rules by physicians based on clinical practice, involving symptoms and laboratory values that indicate flare activation within a specific domain. |
| *Laboratory* | Data extracted from both structured and unstructured sources. Normal ranges are determined according to standard clinical practice guidelines. |
| *Complexity* | Primarily determined by the number and type of involved domains. This categorization is the focus of a dedicated paper that is currently under review. |
| *Therapy Change* | Defined as the adjustment of treatments, either increasing or decreasing intensity among therapy groups. For example, adding an immunosuppressant to hydroxychloroquine is considered a step-up change, while discontinuing bDMARDs is classified as a step-down change. |
| *Treatment* | Extracted through NLP from clinical reports and categorized into major treatment groups. |

Table 2. Comprehensive list of input variables used in model development, including: definition, format (numerical or categorical), and the range type (current contact, last 12 months, and/or patient’s history).

| **Variable Name** | **Definition** | **Format** | **Type** |
| --- | --- | --- | --- |
| Age at contact | Patient's age at the current contact | N | Current |
| Age at baseline | Patient's age at the first recorded contact | N | Past |
| Female gender | Indicates if the patient is female | C | Current |
| Admission | Indicates if the contact is a hospitalization | C | Current, Last, History |
| Outpatient visit | Indicates if the contact is an outpatient visit | C | Current, Last, History |
| Day hospital | Indicates if the contact is a day hospital visit | C | Current, Last, History |
| Delta contacts (days) | Time distance in days from the previous contact | N | Current |
| Total admissions | Number of admissions in the specified period | N | Last |
| Total outpatient visits | Number of outpatient visits in the specified period | N | Last |
| Total day hospitals | Number of day hospitals in the specified period | N | Last |
| Total contacts | Number of contacts in the specified period | N | Last |
| Articular involvement | Presence of involvement of articular domain | C | Current |
| Cutaneous involvement | Presence of involvement of cutaneous domain | C | Current |
| Hematological involvement | Presence of involvement of hematological domain | C | Current |
| Neurologic involvement | Presence of involvement of neurologic domain | C | Current |
| Renal involvement | Presence of involvement of renal domain | C | Current |
| Serosal involvement | Presence of involvement of serosal domain | C | Current |
| Systemic involvement | Presence of involvement of systemic domain | C | Current |
| Vascular involvement | Presence of involvement of vascular domain | C | Current |
| New involvement | Indicates the occurrence of a new organ involvement, not previously present | C | Current, Last, History |
| New articular involvement | Indicates the occurrence of a new articular involvement, not previously present | C | Current, Last, History |
| New cutaneous involvement | Indicates the occurrence of a new cutaneous involvement, not previously present | C | Current, Last, History |
| New hematological involvement | Indicates the occurrence of a new hematological involvement, not previously present | C | Current, Last, History |
| New neurologic involvement | Indicates the occurrence of a new neurologic involvement, not previously present | C | Current, Last, History |
| New renal involvement | Indicates the occurrence of a new renal involvement, not previously present | C | Current, Last, History |
| New serosal involvement | Indicates the occurrence of a new serosal involvement, not previously present | C | Current, Last, History |
| New systemic involvement | Indicates the occurrence of a new systemic involvement, not previously present | C | Current, Last, History |
| New vascular involvement | Indicates the occurrence of a new vascular involvement, not previously present | C | Current, Last, History |
| Total involvements | Total of involved domains. | N | Current |
| Total new involvements | Total of new involved domains. | N | Current, Last, History |
| Articular involvement at baseline | Presence of involvement of articular domain  since the baseline contact | C | History |
| Cutaneous involvement at baseline | Presence of involvement of - cutaneous domain  since the baseline contact | C | History |
| Hematological involvement at baseline | Presence of involvement of hematological domain  since the baseline contact | C | History |
| Neurologic involvement at baseline | Presence of involvement of neurologic domain  since the baseline contact | C | History |
| Renal involvement at baseline | Presence of involvement of renal domain  since the baseline contact | C | History |
| Serosal involvement at baseline | Presence of involvement of serosal domain  since the baseline contact | C | History |
| Systemic involvement at baseline | Presence of involvement of systemic domain  since the baseline contact | C | History |
| Vascular involvement at baseline | Presence of involvement of vascular domain  since the baseline contact | C | History |
| Articular symptom | Presence of a symptom in the articular domain | C | Last, History |
| Cutaneous symptom | Presence of a symptom in the cutaneous domain | C | Last, History |
| Hematological symptom | Presence of a symptom in the hematological domain | C | Last, History |
| Neurologic symptom | Presence of a symptom in the neurological domain | C | Last, History |
| Serosal symptom | Presence of a symptom in the serosal domain | C | Last, History |
| Systemic symptom | Presence of a symptom in the systemic domain | C | Last, History |
| Renal symptom | Presence of a symptom in the renal domain | C | Last, History |
| Vascular symptom | Presence of a symptom in the vascular domain | C | Last, History |
| Articular flare | Presence of a flare in the articular domain | C | Last, History |
| Cutaneous flare | Presence of a flare in the cutaneous domain | C | Last, History |
| Hematological flare | Presence of a flare in the hematological domain | C | Last, History |
| Neurologic flare | Presence of a flare in the neurologic domain | C | Last, History |
| Serosal flare | Presence of a flare in the serosal domain | C | Last, History |
| Systemic flare | Presence of a flare in the systemic domain | C | Last, History |
| Renal flare | Presence of a flare in the renal domain | C | Last, History |
| Vascular flare | Presence of a flare in the vascular domain | C | Last, History |
| C3 consumed | Indicates the reduction of complement C3 (< 90 mg/dL) | C | Current, Last, History |
| C3 normal | Indicates normal or increased levels of complement C3 (≥ 90 mg/dL) | C | Current, Last, History |
| C4 consumed | Indicates the reduction of complement C4 (< 8 mg/dL) | C | Current, Last, History |
| C4 normal | Indicates normal or increased levels of complement C4 (≥ 8 mg/dL) | C | Current, Last, History |
| Albuminuria normal range | Indicates that albumin levels at standard urine examinations are within the normal range (=0 g/L) | C | Current, Last, History |
| Albuminuria out of range | Indicates that albumin levels exceed the normal range (> 0 g/L) | C | Current, Last, History |
| ESR normal range | Indicates that the erythrocyte sedimentation rate is ≤ 30 mm/h | C | Current, Last, History |
| ESR out of range | Indicates that the erythrocyte sedimentation rate is > 30 mm/h | C | Current, Last, History |
| Hemoglobin normal range | Indicates that hemoglobin levels are within the normal range (≥ 11.5 g/dL) | C | Current, Last, History |
| Hemoglobin out of range | Indicates that hemoglobin levels exceed the normal range (< 11.5 g/dL) | C | Current, Last, History |
| Hemoglobin urine normal range | Indicates that hemoglobin levels in the urine are within the normal range (= 0 mg/dL) | C | Current, Last, History |
| Hemoglobin urine out of range | Indicates that hemoglobin levels in the urine exceed the normal range (> 0 mg/dL) | C | Current, Last, History |
| LDH normal range | Indicates that lactate dehydrogenase levels are within the normal range (≤ 250 Ul/L) | C | Current, Last, History |
| LDH out of range | Indicates that lactate dehydrogenase levels exceed the normal range (> 250 Ul/L) | C | Current, Last, History |
| Leukocytes normal range | Indicates that white blood cell count is ≥ 4000/mmc | C | Current, Last, History |
| Low leukocytes count | Indicates that white blood cell count is < 4000/mmc | C | Current, Last, History |
| Lymphocytes normal range | Indicates that lymphocyte count is within the normal range ( ≥ 1000 /mmc) | C | Current, Last, History |
| Low lymphocytes count | Indicates that lymphocyte counts < 1000 /mmc | C | Current, Last, History |
| C-RP normal range | Indicates that C-reactive protein levels are within the normal range (≤ 5 mg/L) | C | Current, Last, History |
| C-RP out of range | Indicates that C-reactive protein levels exceed the normal range (> 5 mg/L) | C | Current, Last, History |
| PLT normal range | Indicates that platelet count is ≥ 100000/mmc | C | Current, Last, History |
| Low PLT count | Indicates that platelet count is <100000 /mmc | C | Current, Last, History |
| Proteinuria normal range | Indicates that protein levels are within the normal range (≤ 500 mg/day) | C | Current, Last, History |
| Proteinuria out of range | Indicates that protein levels exceed the normal range (> 500 mg/day) | C | Current, Last, History |
| Red blood cells count normal range | Indicates that urine red blood cell count is within the normal range (= 0 RBCs/HPF) | C | Current, Last, History |
| Red blood cells count out of range | Indicates that urine red blood cell count exceed the normal range (> 0 RBCs/HPF) | C | Current, Last, History |
| Low complexity | Indicates a low level of disease complexity (as defined in Table 5 of this Appendix) | C | Current, Last, History |
| Medium complexity | Indicates a medium level of disease complexity (as defined in Table 5 of this Appendix) | C | Current, Last, History |
| High complexity | Indicates a high level of disease complexity (as defined in Table 5 of this Appendix) | C | Current, Last, History |
| Low complexity at baseline | Indicates a low disease complexity at the baseline contact | C | History |
| Medium complexity at baseline | Indicates a medium disease complexity at the baseline contact | C | History |
| High complexity at baseline | Indicates a high disease complexity at the baseline contact | C | History |
| Low flare | Indicates the presence of low-complexity flare | C | Last, History |
| Medium flare | Indicates the presence of medium-complexity flare | C | Last, History |
| High flare | Indicates the presence of high-complexity flare | C | Last, History |
| Step-down therapy change | Reduction of therapy intensity between antimalarials, conventional immunosuppressive drug, bDMARDs | C | Current, Last, History |
| Step-up therapy change | Increasing of therapy intensity between antimalarials, conventional immunosuppressive drug, bDMARDs | C | Current, Last, History |
| Total step-down therapy changes | Total number of therapy reductions | N | Last, History |
| Total step-up therapy changes | Total number of therapy intensifications | N | Last, History |
| Hydroxychloroquine | Indicates whether the patient is on hydroxychloroquine therapy | C | Current |
| Immunosuppressant | Indicates whether the patient is on immunosuppressive therapy | C | Current |
| bDMARDs | Indicates whether the patient is on bDMARDs | C | Current |
| Glucocorticoids | Indicates whether the patient is on glucocorticoid therapy | C | Current |
| Prednisone High Dosage (>10mg/day) | Indicates that the patient is on a high dosage of prednisone (greater than 10 mg per day) | C | Current |

Legend. Format: C=Categorical; N=Numerical. Type: Current=about current contact; Last=about last 12 months; History=about the patient’s history. C3: complement fraction 3, C4: complemet fraction 4, ESR: erythrocyte sedimentation rate, C-RP: C reactive protein, PLT: platelet. RBC: red blood cell count, bDMARDs: biological Disease Modifying Anti Rheumatic Drugs.

Table 3. Symptoms Definition: clinical criteria applied to identify symptoms, by domain type.

| **Symptom Domain** | **Laboratory** | **Clinical Manifestations** |
| --- | --- | --- |
| *Articular* | ESR, C-RP | Atralgia, Arthritis, Joint Swelling |
| *Cutaneous* |  | Alopecia, Erythema, Malar Rash, Nodules, Angioedema, Oral Aphtae, Papules |
| *Hematological* | HB, PLT, Lymphocytes, Leukocytes |  |
| *Neurological* |  | Epilepsy, Psychosis, Persistent Headache, Stroke/TIA |
| *Renal* | HB, Proteinuria, Albuminuria, Red Blood Cells Count | Hypertension, Anasarca, Edema |
| *Serosal* | LDH, ESR | Pericarditis, Pleuritis, Pleural/Pericardial Effusion, Ascitis |
| *Systemic* | ESR | Fever > 38° |
| *Vascular* |  | Finger Nodules, Acral Ulcers, Raynaud, Gangrene |

Legend: Hb: hemoglobin, PLT: platelet, ESR: erythrocyte sedimentation rate, C-RP: C reactive protein, LDH: Lactate Dehydrogenase,

Table 4. Flares Definition: clinical criteria and rules applied to identify flare events, by domain type.

| **Flare domain** | **Rules used for flare detection with the NLP pipeline** |
| --- | --- |
| *Articular* | «Arthritis» or «joint swelling» in text mining AND increased C-RP |
| *Cutaneous* | New onset of cutaneous symptoms OR  «worsening» of «malar rash», «erythema» or «cutaneous involvement» in text mining OR  «worsening» or «new onset» of alopecia |
| *Hematological* | Platelets reduction of 20% compared to the previous visit or «platelets reduction» in text mining OR  Decrease in Hb of at least 2 g/dL compared to the previous visit OR  «Haemolitic anemia» in text mining OR  Reduced haptoglobin OR  White blood cell reduction  ≥  50% compared to the previous visit |
| *Neurological* | New onset of epilepsy OR  New onset of stroke or TIA OR  New onset of encephalitis or psychosis |
| *Renal* | 24 hours proteinuria >500 mg (if  ≤  500/mg at previous evaluation) OR  Red blood cells > 5 at urine sediment (if previously normal) OR  New diagnosis of arterial hypertension OR  New onset of lower limb edema OR  Worsening of renal function (=creatinine increase  ≥  30% compared to previous evaluation, if previous creatinine  ≥  1.00 mg/dL) |
| *Serosal* | New onset of pleural effusion OR  New onset of pericardial effusion OR  Chest pain + increased C-RP |
| *Systemic* | Fever>38°C AND decreased C3 and/or C4 |
| *Vascular* | New ulcer or gangrene at upper or lower limbs OR  New onset of vasculitic lesions at upper or lower limbs OR  New onset of deep vein thrombosis or pulmonary embolism |

Table 5. Complexity Definition: clinical criteria and rules applied to categorize involved organs and flare into complexity levels.

|  | **Low** | **Medium** | **High** |
| --- | --- | --- | --- |
| *Complexity* | At least one among: Hematological*, Cutaneous, Serosal, Articular** | One among: Systemic, Articular, Neurological, Vascular, Renal, Hematological*** | Two or more involvements in the domains of the medium complexity box |
| *Flare* | At least one among: Hematological*, Cutaneous, Serosal, Articular** | One among: Systemic, Articular, Neurological, Vascular, Renal, Hematological*** | Two or more flares in the domains of the medium complexity box |

Legend. * Haemolitic anemia and thrombocytopenia<50.000 are not considered as «low»; ** Articular involvement is low only if it does not require immunosuppressant; *** including haemolitic anemia and e thrombocytopenia<50.000
