# Supplementary material for: Prediction of 1-Year Activity in Systemic Lupus Erythematosus: Hierarchical Machine Learning Approach
Source: JMIR Form Res. 2025 Aug 22;9:e70200. doi: 10.2196/70200 (PMC12373299; doi:10.2196/70200)
Supplement: Multimedia Appendix 2 [file formative-v9-e70200-s002.docx]

# Multimedia Appendix 2

Table 1. Input features about the current contact of each patient, overall the dataset visits. Categorical features are reported in percentages, while numerical features present the IQR expressed as “median, (Q1, Q2)”.

| **Feature** | **Overall (5962)** | **Feature** | **Overall (5962)** |
| --- | --- | --- | --- |
| ***Current Contact*** |  | ***Current Contact*** |  |
| Female gender | 89,58% | Albuminuria out of range | 10,37% |
| Age at baseline | 42 (32, 50) | ESR normal range | 33,97% |
| Age at contact | 46 (36, 55) | ESR out of range | 19,94% |
| Outpatient visit | 76,60% | Hemoglobin normal range | 37,47% |
| Day hospital admission | 17,09% | Hemoglobin out of range | 19,54% |
| Admission | 6,66% | Hemoglobin urine normal range | 16,59% |
| Delta contacts (days) | 77 (31, 147) | Hemoglobin urine out of range | 5,85% |
| Articular involvement | 71,80% | LDH normal range | 12,80% |
| Cutaneous involvement | 85,79% | LDH out of range | 2,47% |
| Hematological involvement | 83,55% | Leukocytes normal range | 37,67% |
| Neurologic involvement | 21,00% | Low Leukocytes count | 15,63% |
| Renal involvement | 77,00% | Lymphocytes normal range | 34,95% |
| Serosal involvement | 24,42% | Low Lymphocytes count | 14,88% |
| Systemic involvement | 29,27% | C-RP normal range | 36,11% |
| Vascular involvement | 55,22% | C-RP out of range | 16,03% |
| New articular involvement | 1,83% | PLT normal range | 49,35% |
| New cutaneous involvement | 2,05% | Low PLT count | 2,94% |
| New hematological involvement | 1,63% | Proteinuria normal range | 19,27% |
| New neurologic involvement | 0,57% | Proteinuria out of range | 6,21% |
| New renal involvement | 1,64% | Red blood cells count normal range | 5,03% |
| New serosal involvement | 0,94% | Red blood cells count out of range | 2,80% |
| New systemic involvement | 1,11% | High complexity | 78,61% |
| New vascular involvement | 1,69% | Medium complexity | 16,87% |
| New involvement | 7,88% | Low complexity | 4,51% |
| Total new involvements | 0 (0, 0) | Step-down therapy change | 5,75% |
| Total involvements | 5 (4,6) | Step-up therapy change | 6,57% |
| C3 normal | 27,71% | Hydroxychloroquine | 55,74% |
| C3 consumed | 25,71% | Immunosuppressant | 56,57% |
| C4 normal | 44,77% | bDMARDs | 20,18% |
| C4 consumed | 7,46% | Glucocorticoids | 52,88% |
| Albuminuria normal range | 12,70% | Prednisone High Dosage (>10mg/day) | 7,53% |

Table 2. Input features about the last 12 months of each patient, overall the dataset. Categorical features are reported in percentages, while numerical features present the IQR expressed as “median, (Q1, Q2)”.

| **Feature** | **Overall (5962)** | **Feature** | **Overall (5962)** |
| --- | --- | --- | --- |
| ***Last 12 Months*** |  | ***Last 12 Months*** |  |
| Outpatient visit | 80,53% | Neurologic flare | 0,47% |
| Day hospital | 22,68% | Renal flare | 14,64% |
| Admission | 15,70% | Serosal flare | 0,99% |
| Total outpatient visits | 3 (1, 3) | Systemic flare | 1,34% |
| Total day hospitals | 0 (0, 0) | Vascular flare | 2,11% |
| Total admissions | 0 (0, 0) | C3 consumed | 39,68% |
| Total contacts | 3 (3, 3) | C4 consumed | 13,54% |
| New articular involvement | 5,28% | Albuminuria out of range | 18,47% |
| New cutaneous involvement | 5,97% | ESR out of range | 32,96% |
| New hematological involvement | 4,80% | Hemoglobin out of range | 30,22% |
| New neurologic involvement | 1,69% | Hemoglobin urine out of range | 10,57% |
| New renal involvement | 4,68% | LDH out of range | 5,25% |
| New serosal involvement | 2,73% | Low Leukocytes count | 26,00% |
| New systemic involvement | 3,24% | Low Lymphocytes count | 24,12% |
| New vascular involvement | 4,76% | C-RP out of range | 26,67% |
| New involvement | 20,30% | Low PLT count | 5,27% |
| Total new involvements | 0 (0, 0) | Proteinuria out of range | 11,12% |
| Articular symptom | 7,63% | Red blood cells count out of range | 5,55% |
| Cutaneous symptom | 33,58% | High complexity | 75,03% |
| Hematological symptom | 52,75% | Medium complexity | 20,08% |
| Neurologic symptom | 0,75% | Low complexity | 7,20% |
| Renal symptom | 29,29% | High flare | 1,19% |
| Serosal symptom | 0,94% | Medium flare | 20,41% |
| Systemic symptom | 2,99% | Low flare | 25,83% |
| Vascular symptom | 14,66% | Step-down therapy change | 15,30% |
| Articular flare | 6,02% | Step-up therapy change | 17,43% |
| Cutaneous flare | 23,58% | Total step-down therapy changes | 0 (0, 0) |
| Hematological flare | 29,00% | Total step-up therapy changes | 0 (0, 0) |

Table 3. Input features about the history of each patient, overall the dataset. Categorical features are reported in percentages, while numerical features present the IQR expressed as “median, (Q1, Q2)”.

| **Feature** | **Overall (5962)** | **Feature** | **Overall (5962)** |
| --- | --- | --- | --- |
| ***History*** |  | ***History*** |  |
| Age at baseline | 42 (32, 50) | Hematological flare | 58,69% |
| Outpatient visit | 88,21% | Neurologic flare | 2,21% |
| Day hospital | 41,87% | Renal flare | 39,30% |
| Admission | 55,99% | Serosal flare | 4,78% |
| New articular involvement | 35,64% | Systemic flare | 6,88% |
| New cutaneous involvement | 39,97% | Vascular flare | 10,32% |
| New hematological involvement | 33,28% | C3 consumed | 66,35% |
| New neurologic involvement | 10,77% | C4 consumed | 33,16% |
| New renal involvement | 32,22% | Albuminuria out of range | 53,40% |
| New serosal involvement | 13,85% | ESR out of range | 62,60% |
| New systemic involvement | 20,85% | Hemoglobin out of range | 52,13% |
| New vascular involvement | 31,55% | Hemoglobin urine out of range | 35,06% |
| New involvement | 77,39% | LDH out of range | 18,79% |
| Total new involvements | 0 (0, 0) | Low Leukocytes count | 47,38% |
| Articular involvement at baseline | 34,33% | Low Lymphocytes count | 47,90% |
| Cutaneous involvement at baseline | 43,78% | C-RP out of range | 56,11% |
| Hematological involvement at baseline | 48,64% | Low PLT count | 15,48% |
| Neurologic involvement at baseline | 9,66% | Proteinuria out of range | 23,99% |
| Renal involvement at baseline | 43,14% | Red blood cells count out of range | 20,09% |
| Serosal involvement at baseline | 9,63% | High complexity | 75,03% |
| Systemic involvement at baseline | 7,31% | Medium complexity | 55,12% |
| Vascular involvement at baseline | 21,97% | Low complexity | 34,67% |
| Articular symptom | 24,94% | High complexity at baseline | 32,07% |
| Cutaneous symptom | 68,62% | Medium complexity at baseline | 31,68% |
| Hematological symptom | 74,30% | Low complexity at baseline | 36,25% |
| Neurologic symptom | 2,88% | High flare | 7,83% |
| Renal symptom | 66,24% | Medium flare | 50,86% |
| Serosal symptom | 4,70% | Low flare | 58,19% |
| Systemic symptom | 14,48% | Step-down therapy change | 52,65% |
| Vascular symptom | 35,73% | Step-up therapy change | 60,65% |
| Articular flare | 19,15% | Total step-down therapy changes | 1 (0, 2) |
| Cutaneous flare | 55,42% | Total step-up therapy changes | 1 (0, 1) |
