# Supplementary figures and images for: Prediction of 1-Year Activity in Systemic Lupus Erythematosus: Hierarchical Machine Learning Approach
Source: JMIR Form Res. 2025 Aug 22;9:e70200. doi: 10.2196/70200 (PMC12373299; doi:10.2196/70200)

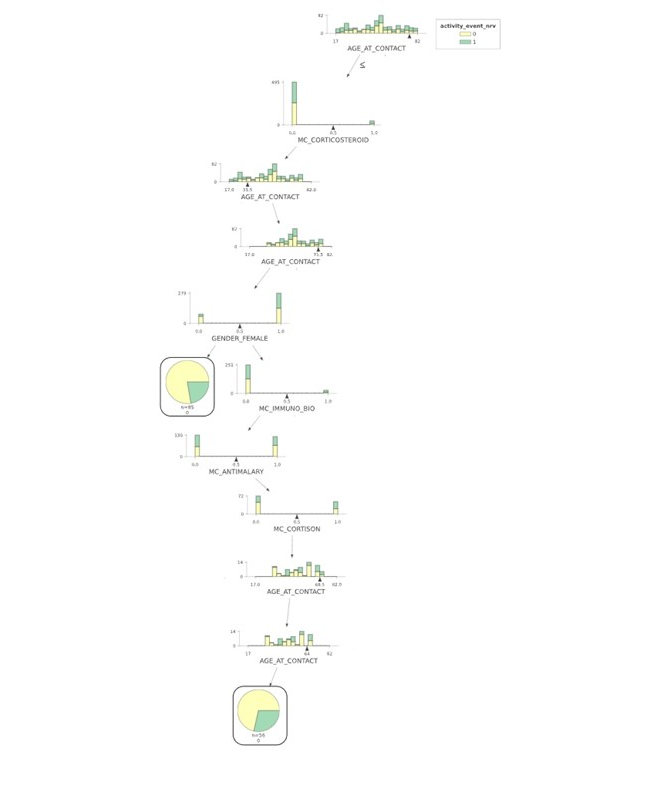

Supplement: Multimedia Appendix 3 [file formative-v9-e70200-s003.png]
