# Supplementary material for: Prediction of 1-Year Activity in Systemic Lupus Erythematosus: Hierarchical Machine Learning Approach
Source: JMIR Form Res. 2025 Aug 22;9:e70200. doi: 10.2196/70200 (PMC12373299; doi:10.2196/70200)
Supplement: Checklist 1 [file formative-v9-e70200-s004.docx]

# Multimedia Appendix 4

In this appendix, we provide the completed checklist for the “Machine Learning Predictive Model for Biomedical Data” guideline, selected as the most appropriate framework for our study. This checklist demonstrates how our work aligns with the recommended reporting standards, supporting transparency, reproducibility, and clarity in accordance with JMIR’s editorial expectations. The official guideline can be accessed at: <https://www.jmir.org/2016/12/e323/>.

Table 2. Checklist.

| **Checklist Item** | **Manuscript Section** | **Comments** |
| --- | --- | --- |
| 1. Nature of Study | Title | We revised the title to clearly state the issue, problem, and method, making the study’s focus clear to the reader. |
| 2. Structured Summary | Abstract | We structured the abstract to include background, objective, method (with data used and approach), results (with performance metrics reported with confidence intervals, and XAI findings), and conclusions (highlighting practical value). |
| 3. Rationale | Introduction | Our Introduction section is already structured to reflect the guidelines, beginning with a clear explanation of the clinical goal and an overview of current practice and existing predictive models. To enhance clarity and alignment with the guidelines, we explicitly titled this subsection "Rationale" within the Introduction. |
| 4. Objectives | Introduction | As with the third point, our Introduction clearly outlines the goal of the study and defines the target of prediction. To enhance the structure and clarity in line with the guidelines, we chose to separate the content into two distinct subsections, where "Objective" clearly states the nature of the study as a predictive modeling task and how this approach can support clinical decision-making. |
| 5. Setting | Methods | We provided details about the clinical setting in the Methods section, specifically in the Data Collection paragraph. This includes the pathology studied (SLE), the facility (Gemelli Hospital), and the cohort selection, which was based on patients with at least one outpatient visit and one hospitalization filtered by ICD-9 codes 710.0 and 695.4. We also describe the selection time-range and the volume of data collected. These elements define the modeling context in terms of clinical setting, selection criteria, and data scale, in line with the guideline’s recommendations. |
| 6. Prediction Problem Definition | Methods | In the Methods section, we clearly define the prediction problem in the Outcome Definition subsection. Here, we explain that the model was developed to predict the occurrence of an activity event within the next 12 months for a given patient. The outcome is explicitly defined as a categorical variable, composed of a combination of clinical events, and we state that the prediction is done per contact, treating each contact independently along the patient’s longitudinal history.  Additionally, in the Main Model subsection, we detail how model performance was evaluated using the AUC metric, which serves as the main quality criterion for assessing prediction success.  Finally, in the Data Collection subsection, we specify that the dataset is a retrospective collection of features related to Lupus, clearly framing the study's design and context in accordance with the guideline’s requirements. |
| 7. Data Preparation | Methods | We address the elements of this guideline across several parts of the Methods section. The Data Collection subsection provide information about the data sources and inclusion criteria, including cohort selection based on ICD-9 codes. This subsection also specifies the number of patients, total contacts, and the timeframe covered by the retrospective dataset.  We added an Ethical Considerations subsection about the ethics statement.  A dedicated Missing Data subsection explains how missing values were handled for both laboratory and treatment variables.  Regarding the input variables, the Input Features subsection explains that a feature engineering process was conducted to structure and standardize the data. Feature selection was performed using a univariate correlation analysis to identify those most related to the prediction outcome, minimizing risk of information leakage. Basic statistics were used to describe the dataset in relation to the outcome, helping to characterize the distribution of features across positive and negative classes (they are reported in Appendix 2). Further statistics are then reported in the Results section, where we highlight the most relevant differences between outcome groups, on the training set. To interpret model behavior, we applied Explainable AI (XAI) techniques, which allowed us to identify and analyze the most impactful predictor variables—this is explained in Methods and further elaborated in the Results section.  Statistics about the cohort are provided in the Data Collection subsection, offering an overview of patient volumes and observation timelines. The Outcome Definition subsection further details the composition and distribution of the activity event used as the prediction target. As for the main model development, the data splitting strategy is described here: 70% of the data was used for training—including cross-validation and feature selection—while 30% was reserved for final evaluation. To avoid data leakage, all contacts from a single patient were assigned to the same set, ensuring proper independence across folds. |
| 8. Model Building | Methods | The feature selection process is described in the dedicated Feature Selection subsection of the Methods, where we explain the criteria used to retain variables, specifically based on their significant association with the outcome. The Outcome Definition subsection reports the distribution of the outcome, which is well balanced across the dataset, ensuring both positive and negative classes are adequately represented.  As also mentioned in the Main Model subsection, class balance was maintained during the splitting process, ensuring that both training and test sets had a comparable distribution of the outcome. This was a key criterion to avoid introducing bias and to preserve model robustness across sets. In this subsection, we also outline the evaluation of several candidate machine learning models through a grid search approach, combined with k-fold cross-validation. The model with the highest AUC was selected as the final predictive model. |
| 9. Final Model and Performance | Results | The Results section presents detailed findings on feature selection and explainability, and model performance. In particular, the XAI analysis identifies the most predictive features influencing the model’s decisions—such as age at contact, therapy changes, laboratory abnormalities, and organ domain involvements. These features offer clinical insights into which factors most strongly contribute to the prediction.  The Risk Stratification subsection further enables the identification of subgroups of contacts with strong or moderate predictive power, as well as a subset of mild-risk predictions, where the main model shows greater uncertainty. To enhance prediction in this latter group, we applied a cascade model trained specifically on mild-risk contacts.  Finally, the Hierarchical Model subsection first presents the performance comparison among the different candidate models, leading to the selection of the main model based on AUC. It then reports the AUC performances of both the main and hierarchical models across different dataset scenarios, demonstrating how the integration of the cascade model improves prediction in more uncertain cases. All results are accompanied by 95% confidence intervals to ensure robust performance assessment. |
| 10. Clinical Implications | Discussion | In the “Discussion” section, we use the feature explainability analysis to bridge model findings with clinical implications. The analysis covers both confirmative results (i.e., which priority features identified through SHAP method are in line with current clinical knowledge) as well as novel features which that may be of help for characterization and early identification of risk profiles in clinical practice. |
| 11. Limitations | Discussion | Within the Limitations subsection, we address the main constraints of the study and link them to potential directions for future work. In particular, we highlight the opportunity to broaden the cohort by including outpatients only, which would allow the model to capture a wider spectrum of disease severity—beyond the current inclusion criteria that require at least one hospitalization. Additionally, we suggest that applying the model in a multicenter setting could further test its generalizability and strengthen its clinical relevance across diverse healthcare contexts. |
| 12. Unexpected Results | Discussion | Throughout the study, intermediate and final results were continuously reviewed and validated through close collaboration between the technical and clinical teams. Beyond the identification of novel characterization features (as discussed above), none of the findings were found to contradict established clinical knowledge or practice |
